# Supplementary material for: Selective breeding enhances coral heat tolerance to marine heatwaves
Source: Nat Commun. 2024 Oct 14;15:8703. doi: 10.1038/s41467-024-52895-1 (PMC11473779; doi:10.1038/s41467-024-52895-1)
Supplement: Supplementary file 1 — Supplementary Information [file 41467_2024_52895_MOESM1_ESM.pdf]

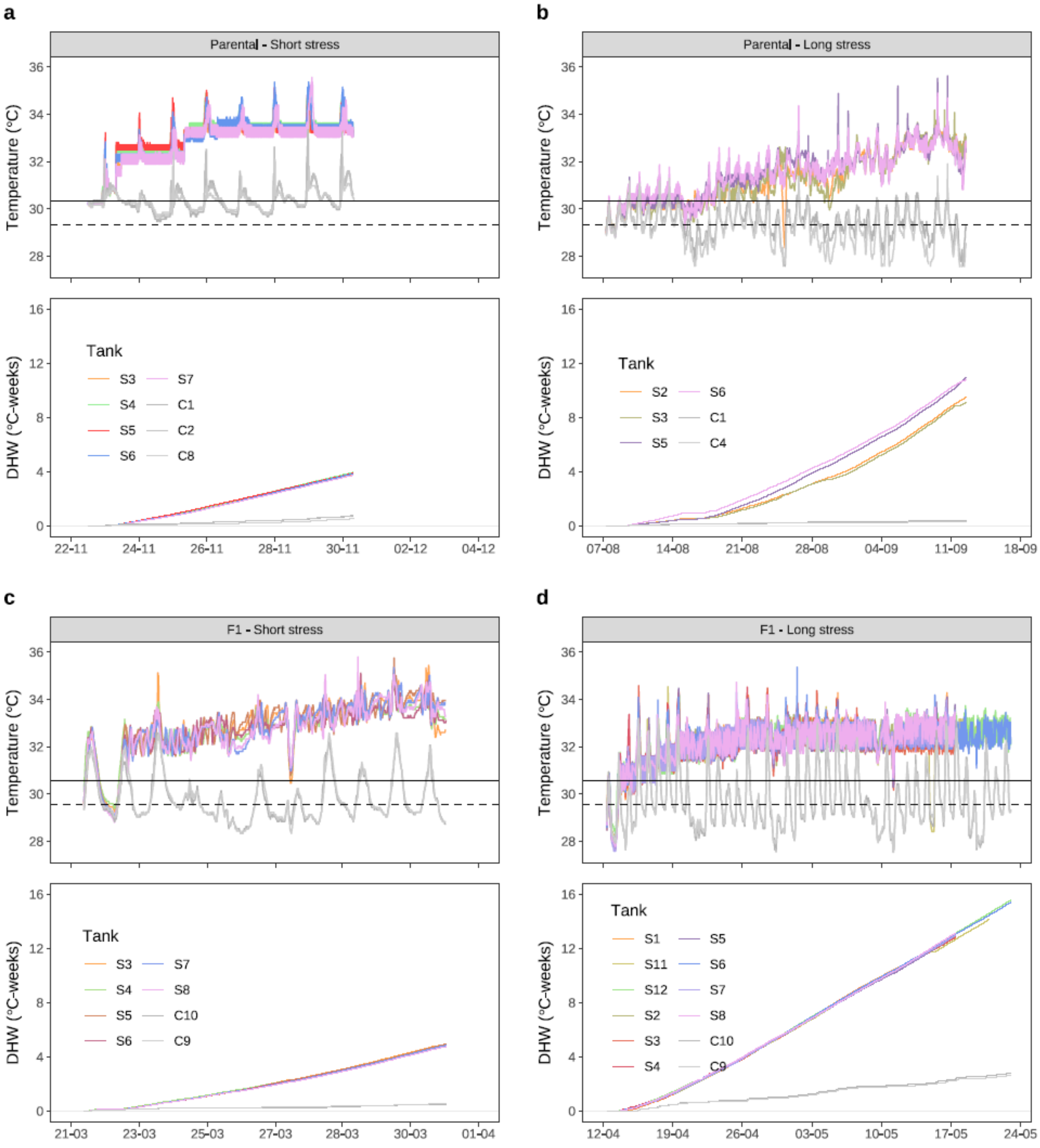

Supplementary Fig. 1. Experimental temperature and heat stress levels.
Temperature stress experiments conducted on source population corals (**a**,**b**) and selectively bred offspring (**c**,**d**). Temperature profiles for replicate heat stress and control tanks are shown in upper panels, and the level of accumulated heat stress measured in Degree Heating Weeks (DHW) is shown in lower panels. Short- and long-stress experiments are shown on the left (**a**,**c**) and right (**b**,**d**), respectively.

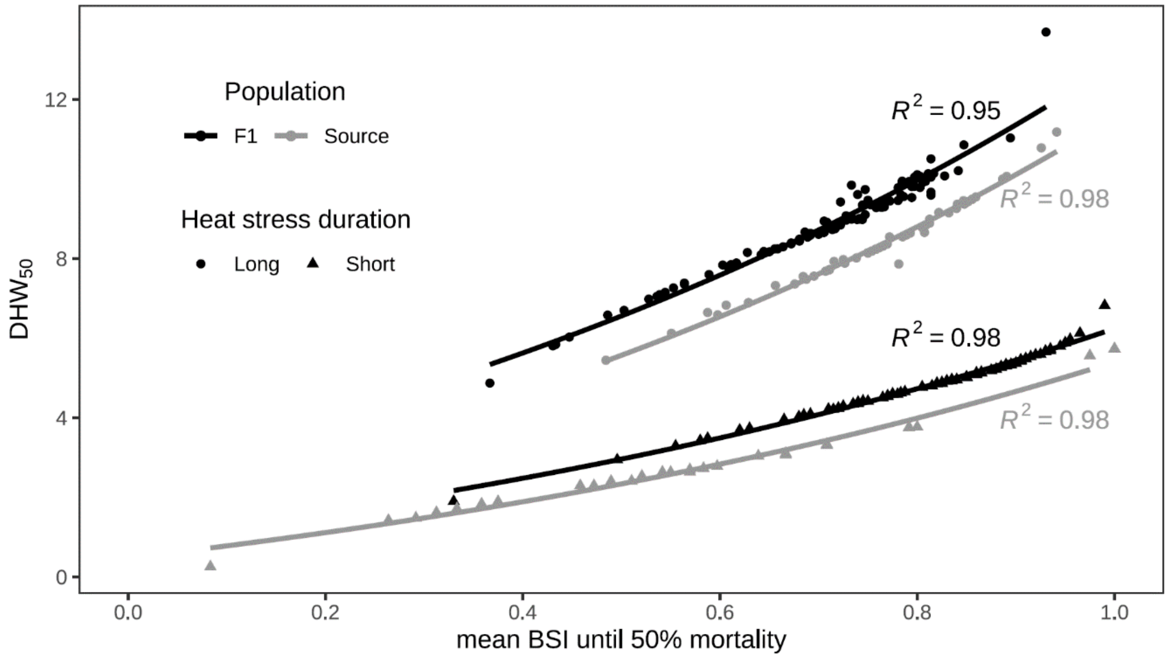

Supplementary Fig. 2. Correlations between mean BSI and predicted DHW tolerance (DHW_{50}).

Comparison of predicted DHW_{50} of each colony versus their mean BSI throughout each exposure (calculated from the first day until 50% mortality of nubbins in the overall experiment). Correlation coefficients (R^2) of log regression models are shown for the short-stress tested source population (grey triangles, n=28 colonies), short-stress tested F1s (black triangles, n=88 colonies), long-stress tested source population (grey circles, n=65 colonies), and long-stress tested F1s (black circles, n=108 colonies).

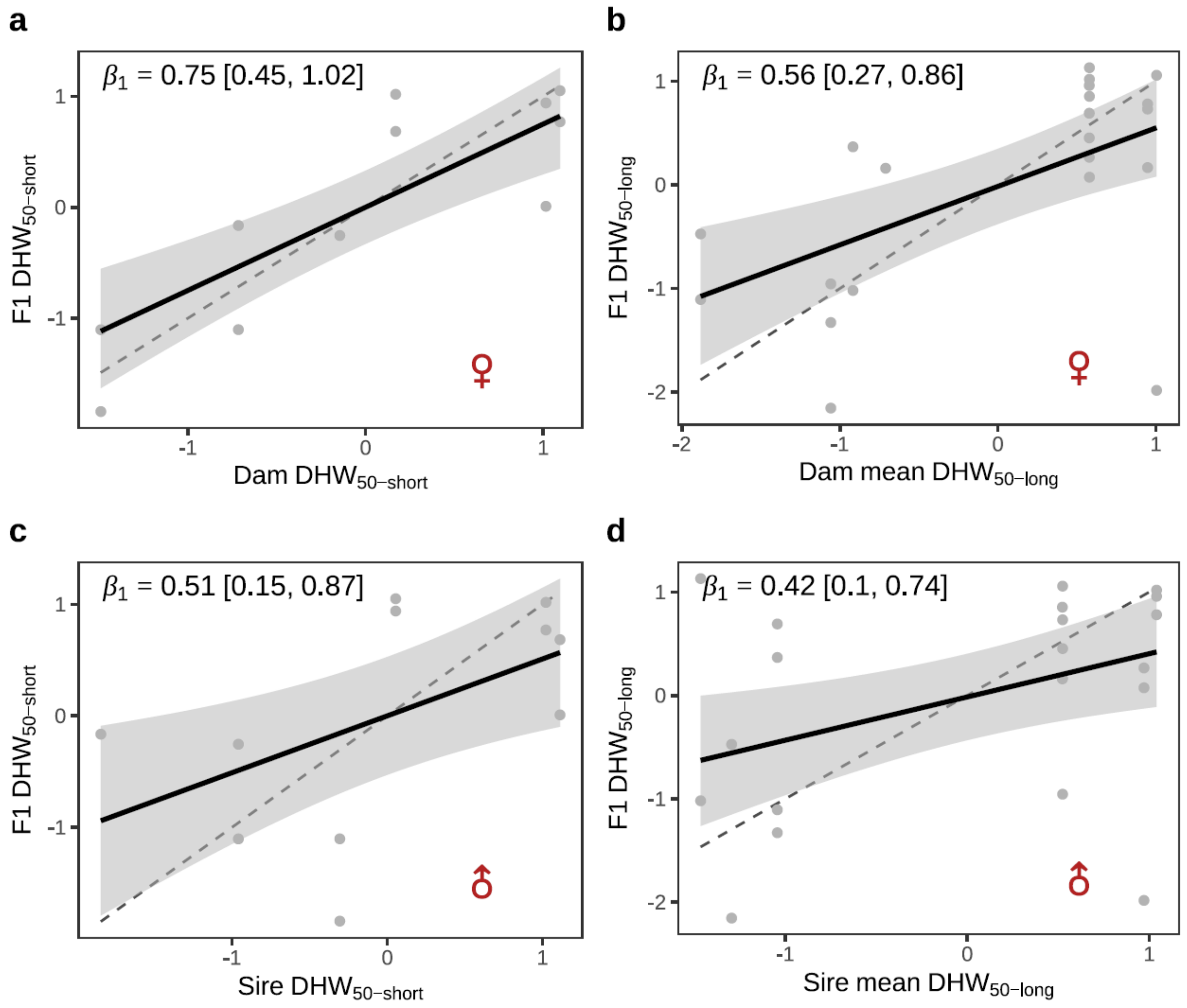

Supplementary Fig. 3: Mother-offspring and father-offspring regressions of short- (a,c) and long-term (b,d) heat stress tolerance.

Heat tolerance of offspring family mean (F1) in relation to the maternal trait value (a,b) or paternal trait value (c,d) for short- (a,c, n=11 families each) and long-term (b,d, n=22 families each) heat stress exposures, based on each colony's ΔDHW_{50}, the heat stress dosage at which the bleaching survival index BSI passes 0.5. The slope is shown as a posterior mean with 95% credible intervals (square brackets) calculated considering random intercepts for each cross. All data are standardised and shown as z scores, such that perfect inheritance of trait values would be represented by a 1:1 relationship (dashed line) between the parent and offspring (F1) heat tolerances. The predicted regression (bold line) and Bayesian 95% credible intervals (shading) are shown.

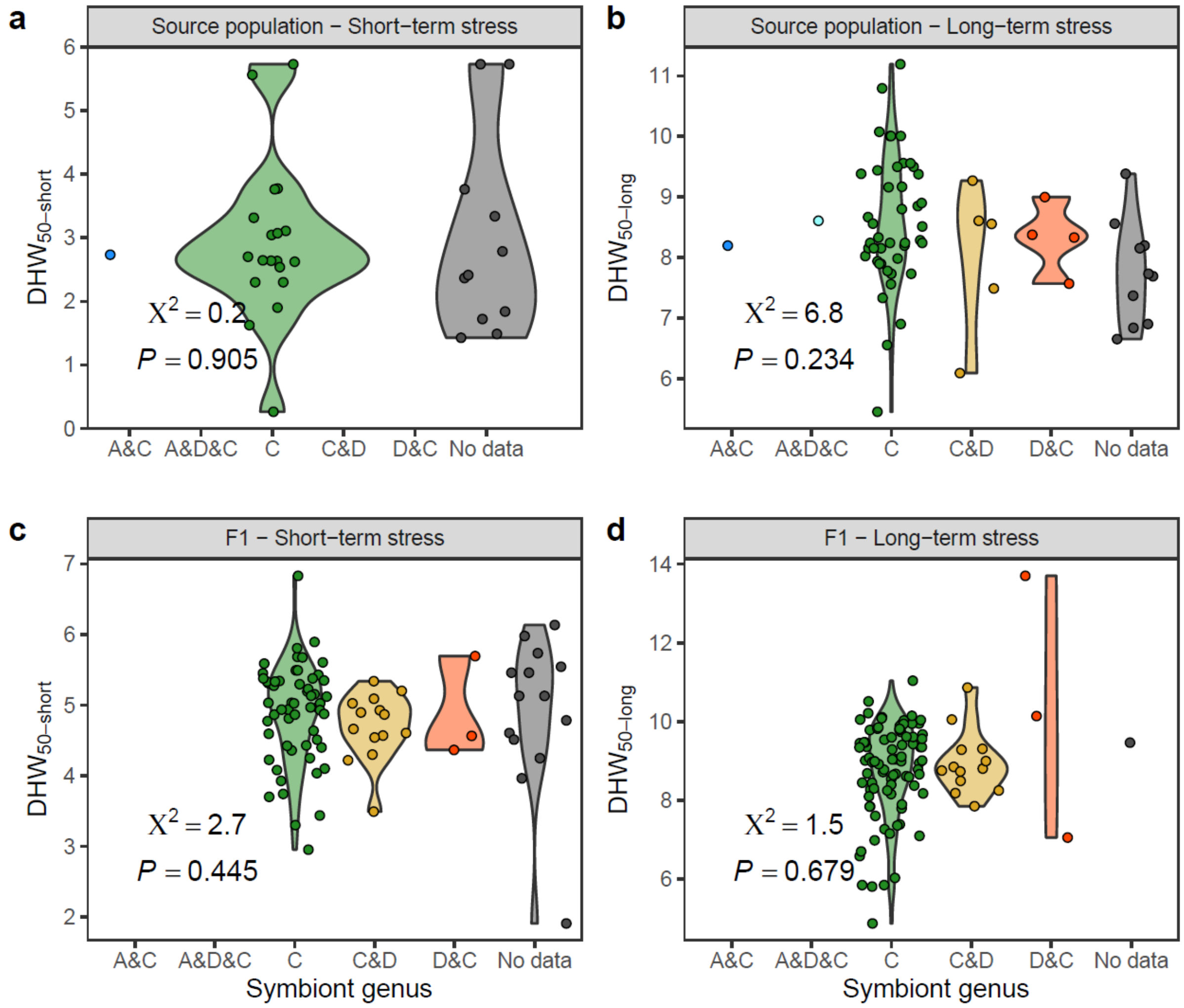

Supplementary Fig. 4: Heat tolerance of source population and selectively bred (F1) coral colonies in relation to symbiont genera.

(**a, b**) Violin plots (rotated kernel density plots) of heat tolerance (in terms of DHW_{50}) of source population colonies in relation to symbiont genera clade A = Symbiodinium, clade D = Durusdinium and clade C = Cladocopium, see [51]. Some minor changes in symbiont community were seen between 2017 and 2018, when the source population was sampled for the short- (**a**, n=31 colonies) and long-term (**b**, n=65 colonies) heat tolerance selection experiments. The majority of source population coral colonies (83%) contained only *Cladocopium* spp. symbionts, typically with C40 dominated ITS2 profiles (see supplementary information for details). Smaller percentages of colonies hosted mixed communities with two or more symbiont genera, ordered by relative abundance: C+D (8%), D+C (6%), with very rare occurrence of *Symbiodinium*: A+C (one colony), and A+D+C (one colony). (**c, d**) Violin plots of DHW_{50} of offspring (F1) in relation to the symbiont genus hosted for the short-stress (**c**, n=88 colonies) and long-stress (**d**, n=104 colonies) experiments. Symbiont composition of the offspring (F1) was broadly similar to the source population, with 84% hosting *Cladocopium* spp. symbionts (mostly C40-dominatedprofiles) and small percentages hosting multi-genus communities (C+D = 13%, D+C = 2%). There were no significant associations between heat tolerance (DHW_{50}) and symbiont community type (Wilcoxon sum ranked test statistics shown in each panel). Relative sequence read abundance and ITS2 profile composition for individual colonies are available on SymPortal (symportal.org) and heat tolerance profiles for each ITS2 type profile are shown in Supplementary data.

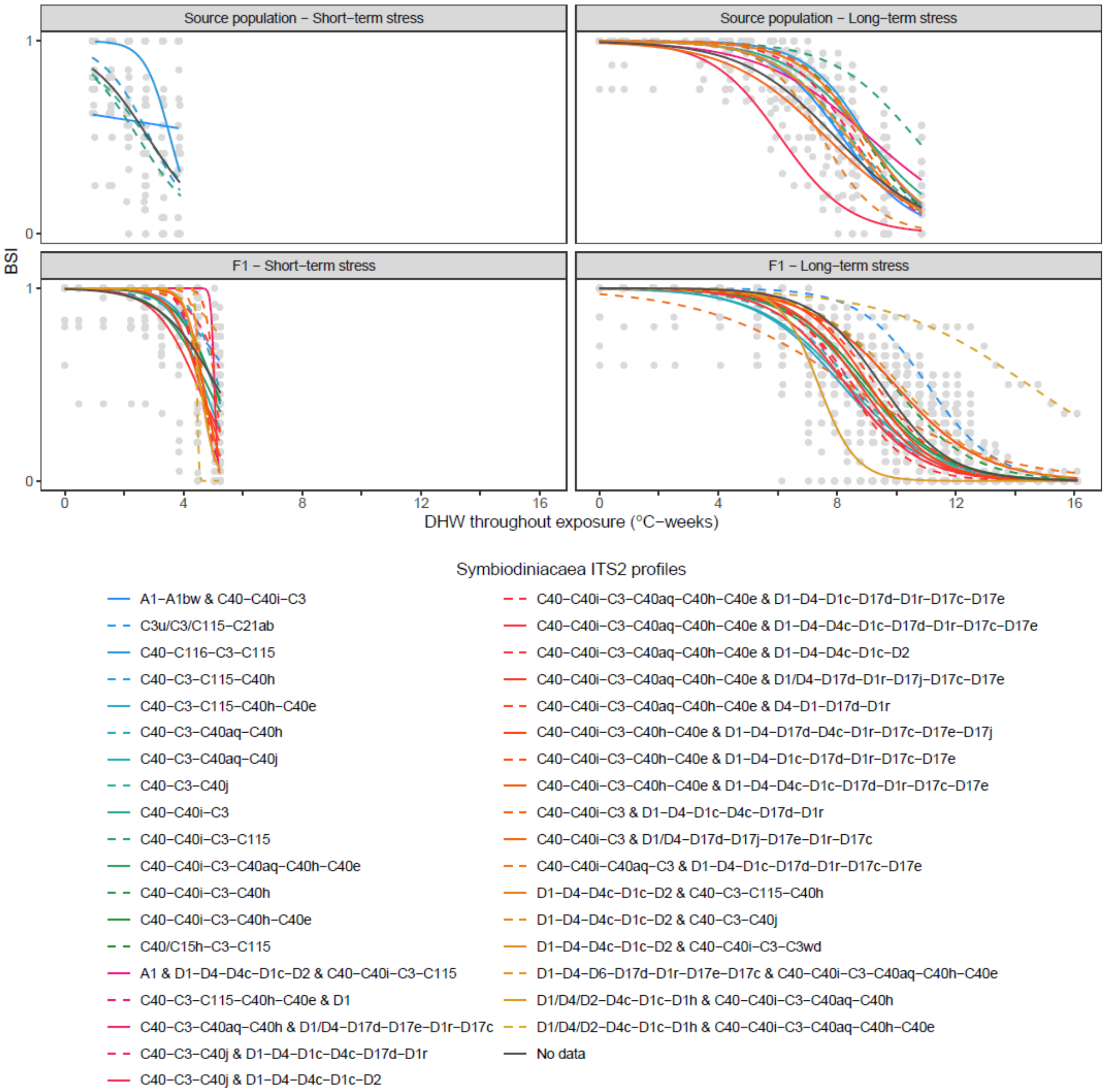

Supplementary Fig. 5. Heat tolerance of source population (i.e., all assayed wild colonies including those selected for breeding) and selectively bred (F1) coral colonies in relation to symbiont ITS2 profiles.

The effect of symbiont community composition on BMI profiles, at the level of individual ITS2 profiles, based on generalised linear mixed effect models with binomial error distribution. Points show BSI values for individual colonies at specific levels of heat stress. Lines indicate the different sets of Symbiodiniaceae ITS2 profiles within colonies, where multiple colonies can share the same set of ITS2 profile). The number of colonies used are 31, 65, 88, and 104, for the short-stress source population, long-stress source population, short-stress offspring (F1), long-stress offspring (F1), respectively. No significant pairwise differences were found among any factor levels. Notably, the lower diversity of ITS2 profiles for short rather than long exposures on source population colonies (top two panels) was likely a function of sampling effort, as the ratio between the number of detected ITS2 profiles compared to the sampling effort is consistently 1:4.

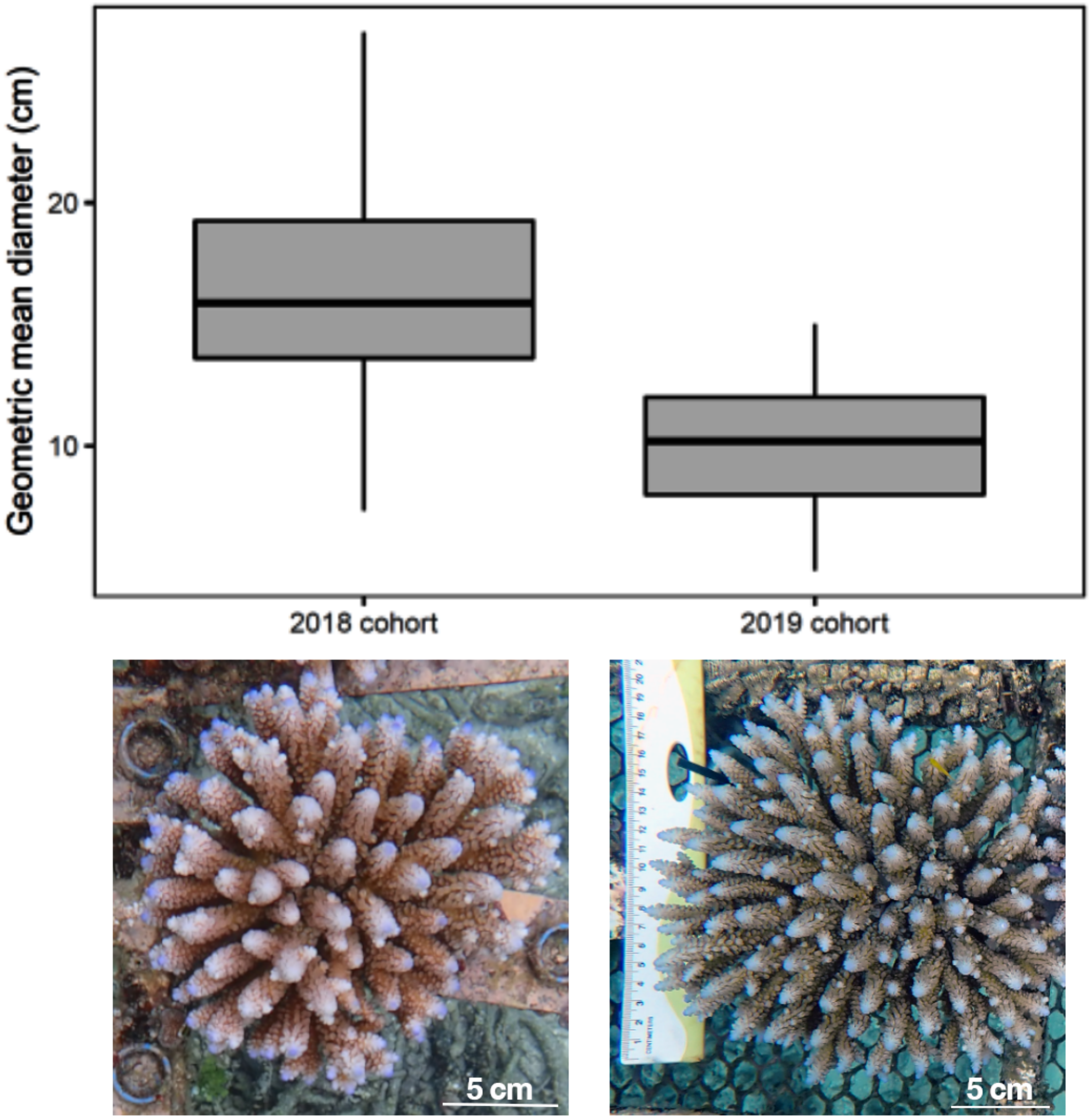

Supplementary Fig. 6. The size of offspring (F1) colonies when sampled in March/April 2022 in terms of geometric mean diameter ($\sqrt{Diameter_{max} \times Diameter_{min}}$) for F1s selected for short-stress tolerance (2018 cohort) and long-stress tolerance (2019 cohort), with example images by cohort shown below (n=170).

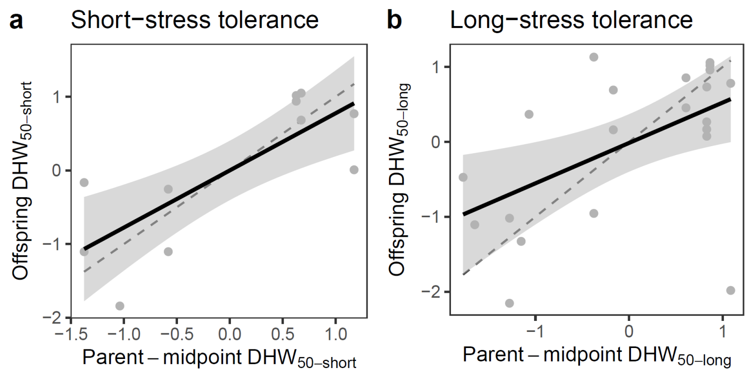

Supplementary Fig. 7. Parent-offspring regressions for (a) short- and (b) long-term heat stress tolerance, using non-informative priors.

(**a, b**) Heat tolerance of offspring (F1) family mean in relation to the mid-parent value for short- (**a**, n=11 families) and long-term (**b**, n=22 families) heat stress exposures, based on each colony's ΔDHW_{50}, the heat stress dosage at which the bleaching survival index BSI passes 0.5. The slope represents the narrow-sense heritability (h^2) of heat tolerance shown as a posterior mean with 95% credible intervals calculated considering random intercepts for each cross. All data are standardised and shown as z scores, such that variance fully attributable to additive genetic effects (h^2 = 1) would be represented by a 1:1 relationship (dashed line) between the parent and offspring heat tolerances. The narrow-sense heritability (h^2) of short- and long-stress heat tolerance was 0.29 (±0.16 SE) and 0.23 (±0.16 SE), respectively, based on a frequentist animal model. This significant heritability is corroborated by the parent-offspring regressions presented here with slightly higher h^2 estimates than the animal model for short- and long-stress heat tolerance of 0.78 (95% credible interval: 0.35–1.2) and 0.54 (95% credible interval: 0.15–0.93), respectively. The predicted regression (bold line) and Bayesian 95% credible intervals (shading) are shown. Non-informative priors for β_1 were used with default Gaussian values (mean of 0 and precision of 0.001).

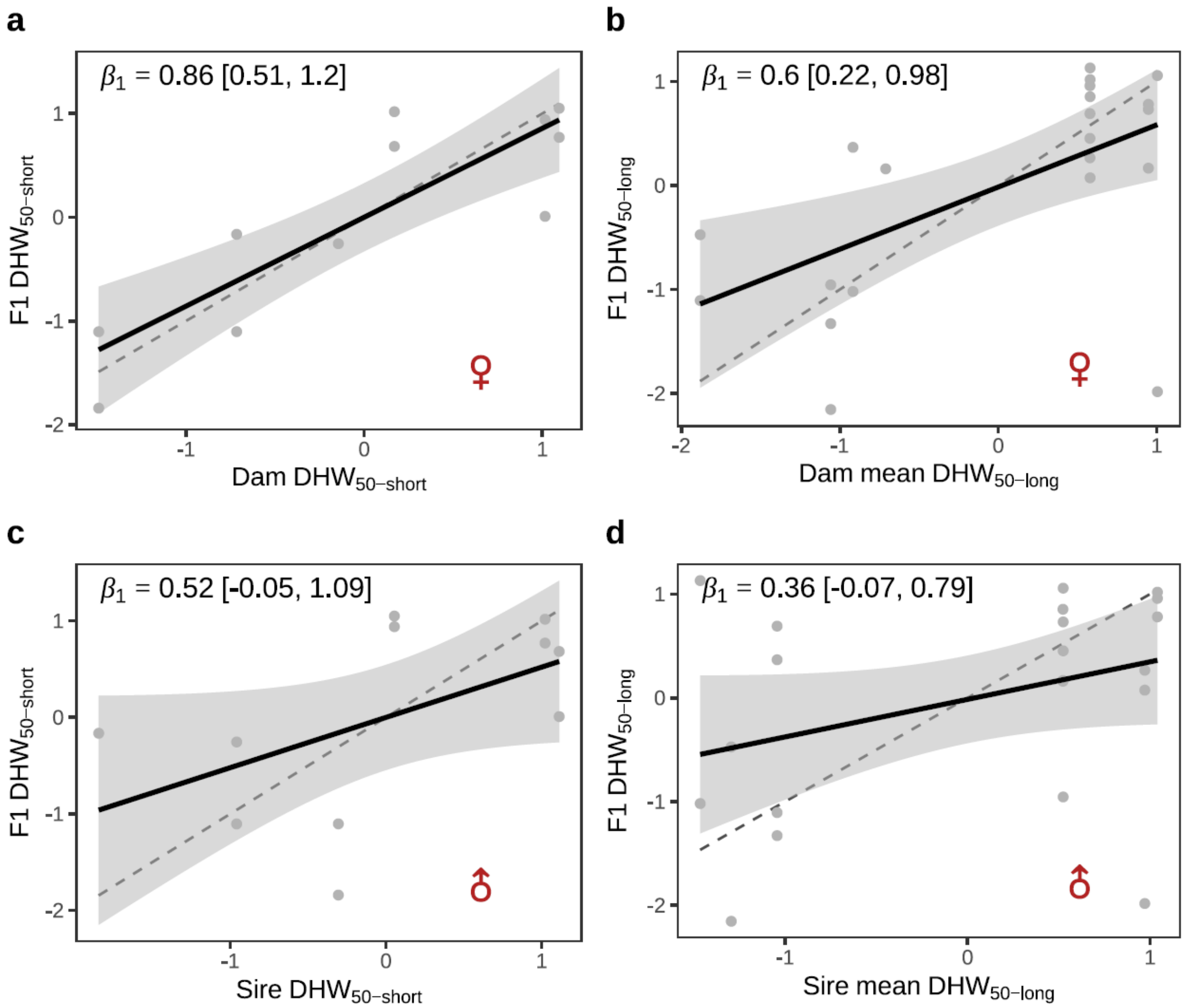

Supplementary Fig. 8. Mother-offspring and father-offspring regressions for short- (a,c) and long-term (b,d) heat stress tolerance, using non-informative priors.

Heat tolerance of offspring family mean (F1) in relation to the maternal trait value (a,b) or paternal trait value (c,d) for short- (a,c, n=11 families each) and long-term (b,d, n=22 families) heat stress exposures, based on each colony's ΔDHW_{50}, the heat stress dosage at which the bleaching survival index BSI passes 0.5. The slope is shown as a posterior mean with 95% credible intervals (square brackets) calculated considering random intercepts for each cross. All data are standardised and shown as z scores, such that perfect inheritance of trait values would be represented by a 1:1 relationship (dashed line) between the parent and offspring heat tolerances. The predicted regression (bold line) and Bayesian 95% credible intervals (shading) are shown. Non-informative priors for the slope, β_1, were used with default Gaussian values (mean of 0 and precision of 0.001).

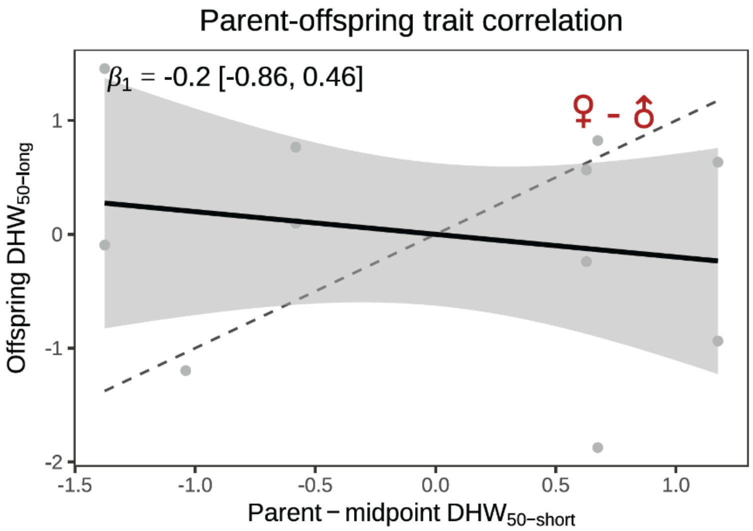

Supplementary Fig. 9. Link between short- and long-term stress tolerance, using non-informative prior.

Offspring produced from parental colonies selected with a short-term heat stress (F1-2018) subjected to a long heat stress exposure. (**c**) Relationship between parent midpoint short-stress tolerance and their offspring's long-stress tolerance based on z-scored data (points, n=11 families), showing no significant genetic correlation (bivariate animal model, r_G = 0.06 (0.66 SE), Z = 0.09, P = 0.90). The regression slope (β_1, the mean and 95% credible intervals of slope posterior distribution), and the predicted regression (mean and 95% credible intervals). A 1:1 relationship (dashed line) would suggest that the same genetic controls are present for both traits. Non-informative priors for the slope, β_1, were used with default Gaussian values (mean of 0 and precision of 0.001).

Supplementary Table 1. Marine heatwave emulation experiment. Associated metadata following[55].

Metadata	Conditions or methods
Coral collection	**Latitude and longitude:**<br>Mascherchur reef: 07°17' 29.3'' N; 134°31' 8.0'' E<br>In situ Nursery: 07°18' 19.8'' N; 134°30' 6.7'' E
	**Collection depth:** between 1 and 5 m depth
	**Collection dates:**<br>2017: November 19th<br>2018: July 27th – August 1st<br>2022 short: March 14th – March 16th<br>2022 long: March 31st – April 9th
	**Coral species:** *Acropora digitifera*
	**Coral morphology:** corymbose
	**Symbiodinaceae for all colonies:**<br>Extended Data Fig. 2017: 20 colonies, 95% have *Cladocopium* only<br>2018: 55 colonies, 80% have *Cladocopium* only<br>2022 short: 74 colonies, 77% have *Cladocopium* only<br>2022 long: 103 colonies, 84% have *Cladocopium* only
	**Acclimation post collection prior to experiment:**<br>2017: 4 days<br>2018: 7 – 11 days<br>2022 short: 5 – 7 days<br>2022 long: 4 – 10 days
Experimental design	**Name of location:** Palau International Coral Reef Center, Palau
	**Bleaching stress temperature period:**<br>2017: November 23rd –November 30th<br>2018: August 8th –September 12th<br>2022 short: March 21st – April 1st<br>2022 long: April 13th – May 23rd
	**System type:** outdoor flow through system
	**No. tanks per treatment:**<br>2017: 5 heat stress & 3 procedural control tanks<br>2018: 4 heat stress & 2 procedural control tanks<br>2022: 6 heat stress & 2 procedural control tanks<br>2022: 10 heat stress & 2 procedural control tanks
	**No. coral genets (colonies) per treatment:**<br>2017: 31 with 7 fragments/colony (4 colonies filtered out)<br>2018: 65 with 6 fragments/colony (1 colony filtered out)<br>2022 short: 88 with 6 fragments/colony (2 colonies filtered out)<br>2022 long: 104 with 6 fragments/colony (no colonies filtered out)
	**No. coral genets (colonies) per tank within treatments:**<br>2017: 17-21 genets per tank<br>2018: 63-65 genets per tank

	2022 short: 36-56 genets per tank<br>2022 long: 64-88 genets per tank
Experimental temperature conditions	**Heat stress temperature above MMM per treatment:**<br>The short-term and long-term heat stress exposures reached approximately +3.5 and +2.5 °C above the locally adjusted climatological baseline (MMM_{adj}: see baseline temperature description below), respectively.
	**Control temperature:**<br>2017: 30.4 ± 0.5 SD °C<br>2018: 29.3 ± 0.7 SD °C<br>2022 short: 29.7 ± 0.9 SD °C<br>2022 long: 29.7 ± 1.2 SD °C
	**Baseline temperature:** The satellite-based CoralTemp MMM was 29.2 °C for both the source population at Mascherchur reef and the offspring in situ nursery which are located in the same 5km grid cell. The local climatological baseline was adjusted to 29.4 °C for Maschechur reef and 29.6 °C for the nursery based on the relationship between satellite sea surface temperatures and *in situ* temperatures[21]. Following convention, the stress baseline for accumulating DHW was set to MMM_{adj} + 1 °C.
	**Temperature ramp-up rate:**<br>Adjustments compared to the MMM_{adj}<br>2017: +1.9 °C on day 1 to 32.5 °C; +1 °C on day 3 to 33.5 °C<br>2018: +0.9 °C on day 1 to ~30.5 °C; +0.5 °C on days 12, 18 & 26 to ~32 °C; +1 °C on day 30 to ~33 °C<br>2022 short: +1.7 °C on day 1 to ~32.5 °C; +0.5 °C on days 3, 5, 7 & 8 to ~33.5 °C<br>2022 long: +0.9 °C on day 1 to ~30.5 °C; +0.5 °C on days 3, 5, 7 & 13 to ~32.5 °C
	**Duration at heat stress temperature:**<br>2017: 7 days<br>2018: 35 days<br>2022 short: 10 days<br>2022 long: 40 days
	**Temperature modulation:** diurnal
Other experimental conditions	**Light conditions:** 400 µmol photons m^{-2} s^{-1}
	**Light cycle:** 12 h:12 h diurnal cycle
	**Water flow velocity:** 7.2 L/hour
	**Tank turnover:** 2 times per day
	**Sea water filtration:** 50µm filtered
	**Sea water source:** natural directly from the reef
	**Salinity:** 35‰
	**Feeding:** natural feeding from plankton in the filtered seawater
