# Supplementary material for: Selective breeding enhances coral heat tolerance to marine heatwaves
Source: Nat Commun. 2024 Oct 14;15:8703. doi: 10.1038/s41467-024-52895-1 (PMC11473779; doi:10.1038/s41467-024-52895-1)
Supplement: Supplementary file 3 — Reporting Summary [file 41467_2024_52895_MOESM3_ESM.pdf]

nature portfolio

Corresponding author(s): Adriana Humanes

Last updated by author(s): 10/09/2024

Reporting Summary

Nature Portfolio wishes to improve the reproducibility of the work that we publish. This form provides structure for consistency and transparency in reporting. For further information on Nature Portfolio policies, see our Editorial Policies and the Editorial Policy Checklist.

Statistics

For all statistical analyses, confirm that the following items are present in the figure legend, table legend, main text, or Methods section.

n/a	Confirmed	
☐	☒	The exact sample size (*n*) for each experimental group/condition, given as a discrete number and unit of measurement
☐	☒	A statement on whether measurements were taken from distinct samples or whether the same sample was measured repeatedly
☐	☒	The statistical test(s) used AND whether they are one- or two-sided<br>*Only common tests should be described solely by name; describe more complex techniques in the Methods section.*
☐	☒	A description of all covariates tested
☐	☒	A description of any assumptions or corrections, such as tests of normality and adjustment for multiple comparisons
☐	☒	A full description of the statistical parameters including central tendency (e.g. means) or other basic estimates (e.g. regression coefficient) AND variation (e.g. standard deviation) or associated estimates of uncertainty (e.g. confidence intervals)
☐	☒	For null hypothesis testing, the test statistic (e.g. *F*, *t*, *r*) with confidence intervals, effect sizes, degrees of freedom and *P* value noted<br>*Give P values as exact values whenever suitable.*
☐	☒	For Bayesian analysis, information on the choice of priors and Markov chain Monte Carlo settings
☐	☒	For hierarchical and complex designs, identification of the appropriate level for tests and full reporting of outcomes
☒	☐	Estimates of effect sizes (e.g. Cohen's *d*, Pearson's *r*), indicating how they were calculated

Our web collection on statistics for biologists contains articles on many of the points above.

Software and code

Policy information about availability of computer code

Data collection	No software was used to collect data
Data analysis	R version 4.0.2

For manuscripts utilizing custom algorithms or software that are central to the research but not yet described in published literature, software must be made available to editors and reviewers. We strongly encourage code deposition in a community repository (e.g. GitHub). See the Nature Portfolio guidelines for submitting code & software for further information.

Data

Policy information about availability of data

All manuscripts must include a data availability statement. This statement should provide the following information, where applicable:

- Accession codes, unique identifiers, or web links for publicly available datasets
- A description of any restrictions on data availability
- For clinical datasets or third party data, please ensure that the statement adheres to our policy

Data availability
The data generated in this study on coral heat tolerance phenotypes, crosses conducted, coral colony sizes, symbiont ITS2 profiles, and temperature experiment conditions have been deposited at https://doi.org/10.25405/data.ncl.22812194. Source data are provided with this paper. All datasets analysed are publicly available as of the date of publication. ITS2 sequences have been archived publicly at NCBI under BioProject 864615 (http://www.ncbi.nlm.nih.gov/

bioproject/864615, accession code PRJNA864615).
Code availability
All datasets generated in this study and original R scripts used for analysis have been deposited at https://doi.org/10.25405/data.ncl.22812194. Processed symbiont community composition can be explored publicly at https://symportal.org.

Research involving human participants, their data, or biological material

Policy information about studies with human participants or human data. See also policy information about sex, gender (identity/presentation), and sexual orientation and race, ethnicity and racism.

Reporting on sex and gender	n/a
Reporting on race, ethnicity, or other socially relevant groupings	n/a
Population characteristics	n/a
Recruitment	n/a
Ethics oversight	n/a

Note that full information on the approval of the study protocol must also be provided in the manuscript.

Field-specific reporting

Please select the one below that is the best fit for your research. If you are not sure, read the appropriate sections before making your selection.

☐ Life sciences ☐ Behavioural & social sciences ☒ Ecological, evolutionary & environmental sciences

For a reference copy of the document with all sections, see nature.com/documents/nr-reporting-summary-flat.pdf

Ecological, evolutionary & environmental sciences study design

All studies must disclose on these points even when the disclosure is negative.

Study description	A field and aquarium study on 288 individual reef-building Acropora digitifera corals to estimate narrow-sense heritability of coral heat tolerance to short and long-term heat stress exposure, as well as an estimate of how much selective breeding could increase offspring heat tolerance.
Research sample	The reef-building coral Acropora digitifera was used as a model species given its widespread distribution and abundance on shallow reefs throughout the Indo-West Pacific. All corals were sourced from a shallow sheltered outer reef in the Republic of Palau (Mascherchur, N 07°17' 29.3''; E 134°31' 8.0''), where A. digitifera is abundant at depths ranging between 0.5 and 4 m. Heat stress experiments to determine colonies heat tolerances and select the parent colonies for brood stock were conducted at the Palau International Coral Reef Center (PICRC) in 2017 and 2018. In April 2018 and March 2019 reproductive and apparently healthy colonies (n = six and eight respectively) with distinctive relative heat tolerances (Fig. 2 a, b) were collected a few days before the full moon of the expected spawning month (April 1, 2018 and March 21, 2019, respectively). The selection of colonies was based on their heat tolerance category (low or high) and whether they were gravid (i.e., whether they contained mature pigmented oocytes) at the time of collection. In 2018 and 2019 two types of crosses were produced: high sire × high dam (HH), and low sire × low dam (LL) (Fig. 2C). Reciprocal crosses were also conducted in 2019: high sire × low dam (HL), and low sire × high dam (LH) (Fig. 2d). Spawning, fertilization, larval rearing, and settlement, and were conducted under laboratory conditions. The resulting F1 offspring were tagged with a cable tie using a colour coded system to identify from which cross it had originated. Colonies were raised in flow-through nursery tanks (ex-situ nurseries) for 13-months for the 2018-F1 and 6-months for the 2019-F1 before being transferred to in-situ nurseries (N 7°18'19.80"N; E 134°30'6.70"E, Fig. 1B) 2.2 km away from Mascherchur reef. Colonies were raised in in situ nurseries until April 2022, when fragments of colonies from each cross were collected to conduct heat stress experiments to estimate their heat tolerances. In April 2022, fragments from 68 colonies corresponding to 11 unique crosses produced in 2018 (F1-2018, Fig. 1c) were exposed to a short-term heat stress (Extended Data Fig. 1c) of similar in duration and intensity to the one used to select their parental colonies (Extended Data Fig. 1a, Extended Data Table 1). Similarly, in May 2022, fragments from 54 colonies corresponding to 21 unique crosses produced in 2019 (F1-2019, Fig. 1d) were exposed to a long-term heat stress (Extended Data Fig. 1d, Extended Data Table 1) equivalent to the one used to select their parental colonies in 2018 (Extended Data Fig. 1b).
Sampling strategy	Visibly healthy colonies were used in all experiments. Mid to large sized colonies were chosen to limit the influence of size on study outcomes, as well as to be able to have sufficient size to remove some fragments without causing damage to the colony. At least six fragments per colony of similar size were excised from the center of each colony. Binomial theory was used to estimate the likelihood of all nubbins from an individual colony either surviving or dying by chance based on the overall mortality rate on a given day. This analysis indicated that at least 5 nubbins in stress tanks from each colony are needed.

Data collection	Heat tolerance: building a tank system, calibrating thermometers, collecting coral fragments, running assay - AH, HM, LL, AE, JB, JG, LB, EB, DB, ES, AW, JC, MS, RT.<br>Symbiont ID: Collecting samples, DNA extraction, PCR, sending for sequencing and bioinformatics - AH, EB, ES, JG, LL, DB, MS and JCB
Timing and spatial scale	Spatial scale: within a single population on Mascherchur reef in Palau<br>Sampling time period: November 2017 to May 2022<br>Sampling time points heat stress experiments for selecting brood stock: short-term (November 2017), long-term (August 2018)<br>Sampling time points heat stress experiments offspring: short-term (April 2022), long-term (May 2022)
Data exclusions	To ensure quality of the data, we removed colonies from the experiment if: (1) the fragment in the control tank died at any stage of the experiment as this could be indicative of handling effects for that colony (n = 4, 1, 2, and 0 colonies in 2017, 2022 short-term, and 2018, 2022 long-term, respectively), (2) fewer than two fragments were alive at the beginning of the experiment after any losses during the acclimation period (n = 1 colony in 2017 short-term, and 0 colonies in the other three experiments)
Reproducibility	The heat stress assay and symbiont ID have been repeated on different sets of colonies in different years, showing similar heat tolerances for this species in each.
Randomization	Tagged coral colonies were located randomly across the home reef. Short-term 2017: Fragments were randomly distributed among five heat stress tanks and three procedural control tanks, ensuring that each colony had at least one fragment in a control tank.<br>Long-term 2018: fragments were randomly distributed among four heat stress tanks and two procedural control tanks, ensuring that each colony had at least one fragment in a control tank and the remaining in independent stress tanks.<br>Short-term and long-term 2022: fragments were randomly distributed among five heat stress tanks and one procedural control tanks, ensuring that each colony had at least one fragment in a control tank and the remaining in independent stress tanks.
Blinding	Fragments were coded with consecutive numbers so that the colony ID was unknown to the observer.

Did the study involve field work? ☒ Yes ☐ No

Field work, collection and transport

Field conditions	No major disturbances occurred during the 5-year study.
Location	Mascherchur reef, Palau. N 07°17' 29.3''; E 134°31' 8.0'
Access & import/export	All efforts were made to collect and export samples in compliance with local, national and international laws. Less than 10% of the volume of each colony was collected from tagged colonies for experimental work. Both national and state permits were obtained prior to any work commencing and all work was done with full collaboration of the Palau International Coral Reef Center. This work was conducted using Koror State permits (018, 032, 034, 037) and Palau National permits (RE-18-13, RE-19-08), and CITES export permits (permit number PW19-111).
Disturbance	The aim of this study was not to impact on the population of corals. Only few fragments were sampled from each coral colony at less than 10% of the colony volume. All other methods were non-invasive. The survival of colonies on the reef after 3 years was comparable to natural survival rates (~11% per year). Offspring colonies are still in the in situ nurseries and are maintained by our group.

Reporting for specific materials, systems and methods

We require information from authors about some types of materials, experimental systems and methods used in many studies. Here, indicate whether each material, system or method listed is relevant to your study. If you are not sure if a list item applies to your research, read the appropriate section before selecting a response.

Materials & experimental systems

n/a	Involved in the study	
☒	☐	Antibodies
☒	☐	Eukaryotic cell lines
☒	☐	Palaeontology and archaeology
☐	☒	Animals and other organisms
☒	☐	Clinical data
☒	☐	Dual use research of concern
☒	☐	Plants

Methods

n/a	Involved in the study	
☒	☐	ChIP-seq
☒	☐	Flow cytometry
☒	☐	MRI-based neuroimaging

Animals and other research organisms

Policy information about studies involving animals; ARRIVE guidelines recommended for reporting animal research, and Sex and Gender in Research

Laboratory animals	This study did not involve laboratory animals
Wild animals	This study involved tagged corals on the reef spawning them in 2018 and 2019, and rearing their offspring in ex situ and in situ nurseries.
Reporting on sex	The species we were working with (Acropora digitifera) is hermaphroditic.
Field-collected samples	Symbiont ID samples: The composition of the symbiont community was identified from one tissue scraping (<1 cm) per colony, stored in ethanol. DNA was extracted using the Qiagen DNeasy blood and tissue kit with overnight proteinase K digestion. Polymerase chain reaction (PCR) was used to amplify DNA extracted from coral tissue and then sent for ITS2 sequencing.
Ethics oversight	The research presented here adhered to the ethical standards consistent with Newcastle University. No ethical approval is needed to work with corals.

Note that full information on the approval of the study protocol must also be provided in the manuscript.
